# Neuroprotective Activity of Oligomeric Stilbenes from Alpha Grape Stems in In Vitro Models of Parkinson’s Disease

**DOI:** 10.3390/ijms26062411

**Published:** 2025-03-07

**Authors:** Evgeny A. Pislyagin, Darya V. Tarbeeva, Ekaterina A. Yurchenko, Ekaterina S. Menchinskaya, Tatiana Y. Gorpenchenko, Natalya D. Pokhilo, Anatoly I. Kalinovskiy, Dmitry L. Aminin, Sergey A. Fedoreyev

**Affiliations:** 1 G.B. Elyakov Pacific Institute of Bioorganic Chemistry, Far Eastern Branch of the Russian Academy of Sciences, Vladivostok 690022, Russia; tarbeeva1988@mail.ru (D.V.T.); eyurch@piboc.dvo.ru (E.A.Y.); ekaterinamenchinskaya@gmail.com (E.S.M.); pokhilo1952@mail.ru (N.D.P.); kaaniw@piboc.dvo.ru (A.I.K.); daminin@piboc.dvo.ru (D.L.A.); fedoreev-s@mail.ru (S.A.F.); 2 Federal Scientific Center of the East Asia Terrestrial Biodiversity, Far Eastern Branch of the Russian Academy of Sciences, Vladivostok 690022, Russia; gorpenchenko@biosoil.ru; 3 Department of Biomedical Science and Environmental Biology, Kaohsiung Medical University, No. 100, Shin-Chuan 1st Road, Sanmin District, Kaohsiung City 80708, Taiwan

**Keywords:** oligomeric stilbenes, Parkinson’s disease, neuroprotective activity, oxidative stress, reactive oxygen species, antioxidant mechanisms, neuro-2a cells

## Abstract

This study investigated the neuroprotective activity of oligomeric stilbenes (OSs) derived from Alpha grape stems in various in vitro models of Parkinson’s disease (PD). Using neurotoxin-induced cellular models, including 1-methyl-4-phenylpyridine (MPP+), paraquat (PQ), 6-hydroxydopamine (6-OHDA), and rotenone, we screened the cytoprotective effects of ampelopsin A (**1**), ε-viniferin (**2**), vitisin D (**3**), vitisin A (**4**), α-viniferin (**5**), *trans*-vitisin B (**6**), *cis*-vitisin B (**7**), and melanoxylin A (**8**). The results demonstrate that certain stilbenes significantly enhanced cell viability and reduced reactive oxygen species (ROS) levels in neurotoxin-treated Neuro-2a cells. Notably, vitisin A and *trans*-vitisin B exhibited promising neuroprotective properties by decreasing mitochondrial ROS and cardiolipin peroxidation. This study highlights the potential of these compounds in mitigating oxidative stress and inflammation associated with PD. Additionally, we provided new insights into the antioxidant mechanisms of these stilbenes, including their direct ROS-scavenging abilities. Our findings contribute to the understanding of oligomeric stilbenes as potential therapeutic agents for the prevention and treatment of neurodegenerative diseases, particularly those associated with oxidative damage. Further research is warranted to explore its clinical applications and underlying mechanisms of action.

## 1. Introduction

A change in the age structure of the human population has led to an increase in the prevalence of age-related diseases such as oncological, cardiovascular, and neurodegenerative disorders. As such, in three decades, the world prevalence of Parkinson’s disease has increased dramatically from 0.90 cases per 1000 in 1980–1989 and 1.38 cases per 1000 in 1990–1999 to 1.18 cases per 1000 in 2000–2009 and 3.81 cases per 1000 in 2010–2023 [1]. Parkinson’s disease was ranked as the 45th cause of death globally in 1990 and rose to 32nd in 2021 [2]. There is also a bias of men suffering from this disease, although women are also susceptible to it. The highest prevalence of this disease occurs at the age of 70+ years; however, in the last decade, there has been an increase in the incidence among able-bodied and socially active people in the 40–49 age group and, especially, in the 50–69 age group [3], which is socially significant and requires serious attention.

PD is characterized by a progressive loss of dopaminergic neurons (DAn) in the substantia nigra pars compacta (SNpc) and a loss of dopamine in the striatum (DA) [4], leading to motor dysfunction including tremors, slowness of movement, rigidity, and gait disturbance. In addition, PD patients often experience non-motor symptoms, such as depression, cognitive impairment, sleep disturbances, and olfactory disturbances [5]. PD is a chronic and progressive condition, and while current treatments can alleviate symptoms, they are unable to slow or halt disease development [6]. Consequently, it is imperative to create novel strategies for treating PD with the aim of enhancing patients’ quality of life and decelerating disease advancement.

The molecular basis of Parkinson’s disease (PD) is still poorly understood, but evidence supports the involvement of inflammation in its progression. Neurohistological and neuroimaging studies show the presence of neuroinflammatory processes. Changes in inflammatory markers and immune cell populations can exacerbate neurodegeneration [7].

In addition, genetic factors, exposure to viruses and pesticides, and changes in the gut microbiota contribute to the pathogenesis of PD. Complex gene–environment interactions, combined with an aging immune system, create conditions for the development of the disease [8].

The exact cause of PD is not known, but heavy metals (iron, mercury, manganese, copper, and lead) are associated with its progression. Chronic exposure to these metals activates proinflammatory cytokines, leading to neuronal loss. Metals disrupt redox homeostasis, causing oxidative stress and cellular damage which may contribute to dopaminergic neurodegenerative disorders [9].

The proposed pathogenic mechanisms of PD include increased alpha-synuclein (*SNCA*) expression and α-synuclein aggregation, altered endosomal–lysosomal trafficking, lysosomal dysfunction, neuroinflammation, cell-to-cell spread, and mitochondrial dysfunction [10]. Multiple *PRKN* (parkin RBR E3 ubiquitin protein ligase), *PINK1* (PTEN-induced kinase 1), and *LRRK2* (Leucine-Rich Repeat Kinase 2) gene mutations or environmental factors lead to changes in mitochondrial function, including reduced complex I activity, increased reactive oxygen species, abnormal calcium homoeostasis, and reduced mitochondrial ATP production. Therefore, antioxidants used alone or in combination therapy are promising for the prevention of PD. In 2021, phase I clinical trials of intranasal reduced glutathione therapy (NCT02324426) and two herbal drugs Win-1001X (NCT04220762) and hypoestoxide (NCT04858074) were conducted [11]. In 2022, the clinical effectiveness of the herbal extract DA-9805 [9] containing mitochondria-targeted saikosaponin A, paeonol, and imperatorin [10] was investigated (NCT03189563), and a weak positive effect of DA-9805 on the motor function of patients was detected. This is a very rare case of any effectiveness, as other antioxidants have not shown significant results owing to their low bioavailability [12]. Nevertheless, the strategy of using antioxidants against PD remains relevant because long-term intake of some dietary antioxidants helps reduce the risk of morbidity [13].

Stilbenes are a group of natural compounds found in many plant species and have recently been the focus of much research in the fields of medicine and chemistry. Oligomeric stilbenes (OSs) are polyphenolic compounds consisting of two or more resveratrol or piceatannol moieties. A wide range of natural stilbene derivatives has been identified, with structures ranging from monomers to octamers and with different substituents at different positions, such as glycosyl, hydroxyl, methyl, and isopropyl groups [14]. Some well-known stilbenes include monomeric resveratrol, pterostilbene, and oligomeric stilbenes such as viniferins and vitisins [15]. OSs have a wide range of biological properties, including antioxidant, anti-inflammatory, antimicrobial, and anticarcinogenic properties [16,17]. The antioxidant activity of OS is due to its ability to neutralize ROS, which can damage cellular structures and cause oxidative stress [18]. Several studies have shown that OSs can protect neurons from oxidative stress induced by various factors such as MPP+ [19]. Furthermore, OSs have been shown to penetrate the blood–brain barrier (BBB) and accumulate in brain tissue, making them potentially useful for the treatment of PD [20].

*Vitis vinifera* L. (grape) is an important natural source of stilbenes and belongs to the plant family Vitaceae. This species contains more than 60 stilbenoids, including monomers, such as *trans*-resveratrol and piceatannol, as well as oligomers that are usually in the *trans* configuration [21]. Each grape variety has a unique composition of polyphenolic compounds [22], and thus it is advisable to study them in detail. Previously, we reported the composition of the main oligomeric stilbenes from the stems of Alpha grapes and the cell-free radical scavenging activity of isolated (+)-*cis*-vitisin A, *trans*-ε-viniferin, and *trans*-resveratrol [23].

The aim of this study was to isolate OSs from Alpha grape stems and investigate their cytoprotective potential in neurotoxin-induced Parkinson’s disease models in Neuro-2a cells.

## 2. Results

### 2.1. Isolation and Structure Determination of Compounds

**Figure 1:**
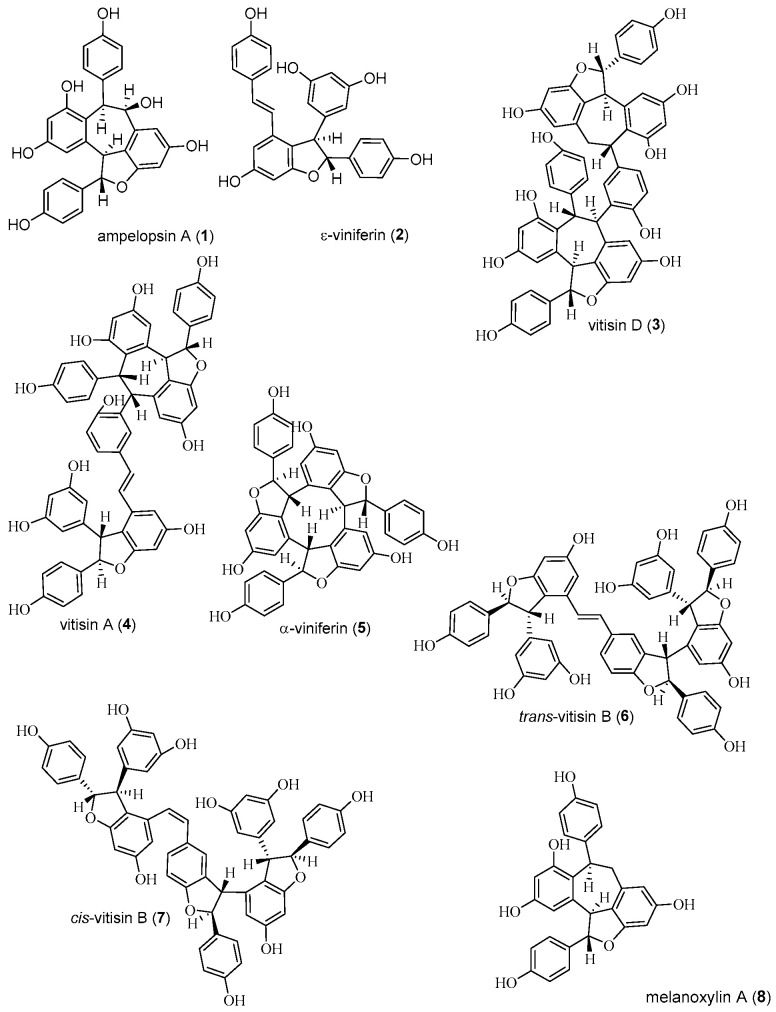
Structures of OSs isolated from Alpha grape stems.

Eight OSs were isolated from Alpha grape stems, and they were identified as ampelopsin A (**1**), ε-viniferin (**2**), vitisin D (**3**), and vitisin A (**4**), as well as α-viniferin (**5**), *trans*-vitisin B (**6**), *cis*-vitisin B (**7**), and melanoxylin A (**8**) (Figure 1) by comparison with published spectral data (Supplementary Materials) [24,25,26].

Ampelopsin A (**1**), ε-viniferin (**2**), vitisin D (**3**), and vitisin A (**4**) have previously been isolated from Alpha grape stems [23]. In this study, we isolated OS **1**–**4** and α-viniferin (**5**), *trans*-vitisin B (**6**), *cis*-vitisin B (**7**), and melanoxylin A (**8**) as individual compounds from Alpha grape stems.

### 2.2. Antioxidant Activity of Stilbenes **1**–**8**

The antioxidant activity of the OSs was tested using two cell-free assays, DPPH (2,2-diphenyl-1-picrylhydrazyl) and FRAP (Ferric-Reducing Antioxidant Power Assay), and the data are presented in Table 1.

The DPPH-radical scavenging activity of compounds **1**–**4** was determined previously [23] and the data are presented in Table 1 for comparison with the DPPH radical scavenging activity of **5**–**8**. ε-Viniferin (**2**), vitisin D (**3**), and vitisin A (**4**) exhibited the most significant DPPH scavenging effects, with IC_50_ values of 63.0, 62.7, and 59.1 µM, respectively. However, these OSs were less active compared to quercetin. The other tested compounds possessed weak antiradical activities and ferric-reducing powers (Table 1).

ε-Viniferin (**2**) and melanoxylin A (**8**) showed the most significant effect in the FRAP assay (Table 1). Notably, melanoxylin A (**8**) did not possess significant DPPH scavenging activity. In the FRAP assay, all the OSs were significantly more effective than quercetin.

All the OSs were less active in the DPPH assay than in the FRAP assay (Table 1).

### 2.3. Effect of OSs on the Viability of MPP+-Treated Neuro-2a Cells

**Figure 2:**
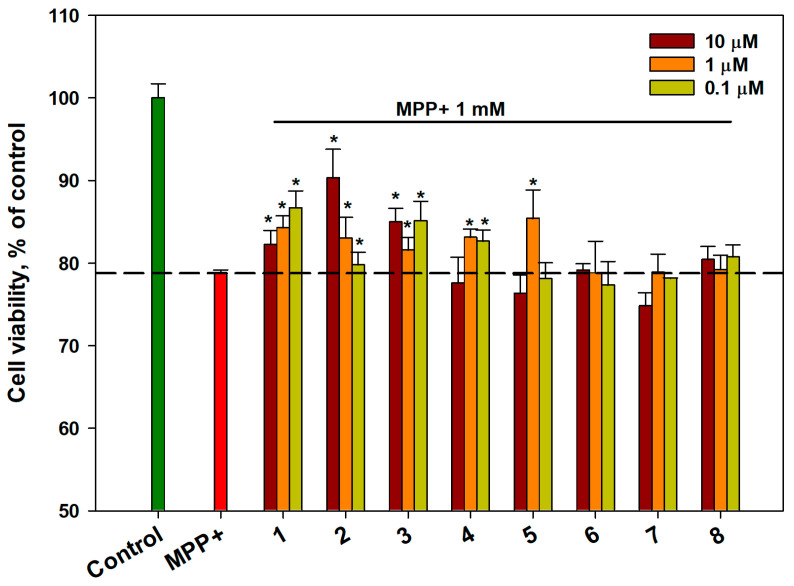
The effect of OSs on the viability of MPP+-treated Neuro-2a cells. Each bar represents the mean ± SEM of three independent replicates. (*) denotes a significant difference (*p* < 0.05) compared to the MPP+-treated cells. The dashed line indicates the level of positive control. The green bar represents the control, while the red bar represents the positive control. Differences between the control and MPP+-treated cells were considered significant.

The influence of OS **1**–**8** on the viability of cells in the MPP+-induced neurotoxicity model was investigated, and the data are presented in Figure 2. MPP+ at a concentration of 1 mM decreased the viability of Neuro-2a cells by 21–22%. The pretreatment of cells with compounds **6**–**8** had no significant effect on the viability of MPP+-treated Neuro-2a cells. Compounds **1**–**5** weakly enhanced cell viability at different concentrations. Compound **2** at a concentration of 10 μM was the most effective in this test and increased cell viability by 14% (Figure 2).

### 2.4. Effect of OSs on PQ-Treated Neuro-2a Cells

**Figure 3:**
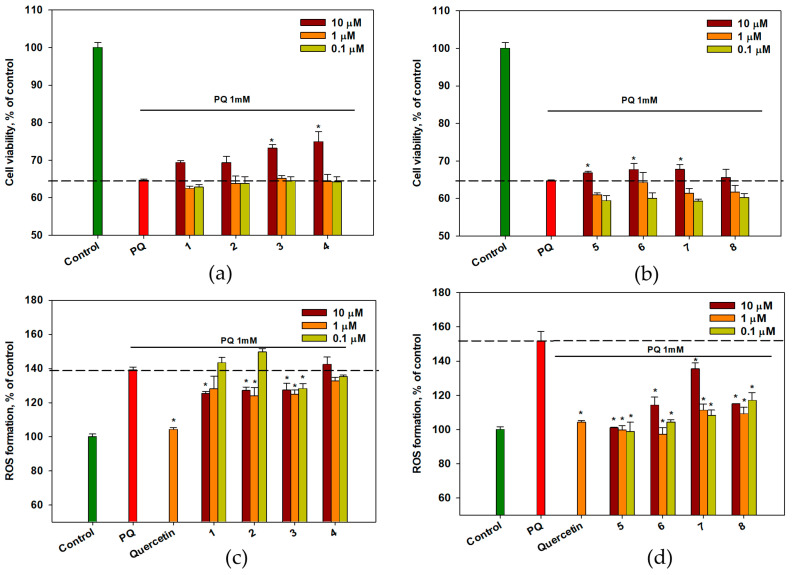
The effect of OSs on cell viability (**a**,**b**) and ROS levels (**c**,**d**) in PQ-treated Neuro-2a cells. Each bar represents the mean ± SEM of three independent replicates. (*) indicates *p* < 0.05, compared to cells treated with PQ. The dashed line indicates the level of positive control. The green bar represents the control, while the red bar represents the positive control. Differences between the control and PQ-treated cells were considered significant.

The influence of OSs **1**–**8** on the viability of cells in a PQ-induced neurotoxicity model was investigated, and the data are presented in Figure 3a,b. PQ at a concentration of 1 mM decreased the viability of Neuro-2a cells by 32–33%. The pretreatment with OSs **3**–**7** at 10 µM increased the viability of PQ-treated cells by 3–11.5%, Compound **4** at a concentration of 10 μM was the most effective. Compounds **1**, **2**, and **8** exhibited no discernible effects on the viability of PQ-treated Neuro-2a cells in this study.

Moreover, the intracellular levels of ROS in PQ-treated cells were measured. The data are presented in Figure 3c,d. PQ significantly increased the intracellular ROS levels by 40%. Surprisingly, OSs **1**–**3** and **5**–**8** markedly diminished ROS levels in PQ-treated cells (Figure 3c,d). Compound **1** at a concentration of 10 μM decreased ROS levels by 10%, and compound **2** at concentrations of 1 and 10 μM decreased ROS levels by 15% and 10%, respectively; however, both compounds had no effect on the viability of PQ-treated cells. At all concentrations, compound **3** decreased the ROS level by 10–12%. Compound **4** was not effective in this test, even though it enhanced the viability of PQ-treated cells.

Compounds **5** and **6** were the most efficacious in this assay, and at 1 μM they both reduced ROS levels by 34% and 36%, respectively, comparable to quercetin (Figure 3d). In addition, compounds **7** and **8** at all concentrations decreased intracellular ROS levels by 10–28%. 

### 2.5. Effect of OSs on 6-OHDA-Treated Neuro-2a Cells

**Figure 4:**
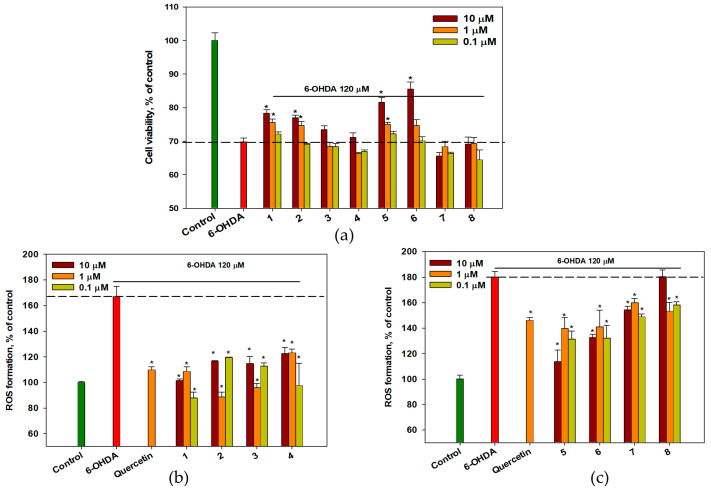
The effect of OSs on viability (**a**) and intracellular ROS levels (**b**,**c**) in Neuro-2a cells treated with 6-OHDA. Each bar represents the mean ± SEM of three independent replicates. (*) indicates *p* < 0.05, compared with cells treated with 6-OHDA. The dashed line indicates the level of positive control. The green bar represents the control, while the red bar represents the positive control. The difference between the control and 6-OHDA-treated cells was considered significant.

The effects of OSs **1**–**8** on cell viability in the 6-OHDA-induced neurotoxicity model were assessed, and the results are presented in Figure 4a. 6-OHDA at a concentration of 120 μM decreased Neuro-2a cell viability by 29–30%. Compounds **3**, **4**, **7**, and **8** had no influence on the viability of 6-OHDA-treated cells. At the same time, OSs **1** and **2** increased the viability of 6-OHDA-treated cells by 12.3% and 10.2%, respectively (*p* < 0.05). Compounds **5** and **6** increased the percentage of living Neuro-2a cells following 6-OHDA treatment by 15.2% and 20.8%, respectively, compared with cells treated with 6-OHDA alone (*p* < 0.05).

6-OHDA caused a significant increase in intracellular ROS levels in Neuro-2a cells by 67–80% and all OSs at all tested concentrations significantly prevented it, similar to quercetin (Figure 4b,c). Compounds **1** and **2** at concentrations of 0.1 and 1 μM, respectively, exhibited the highest efficacy in this assay, decreasing ROS levels by 47.4% (0.1 μM) and 47.1% (1 μM), respectively.

### 2.6. The Effect of OSs on Neuro-2a Cells Treated with Rotenone

**Figure 5:**
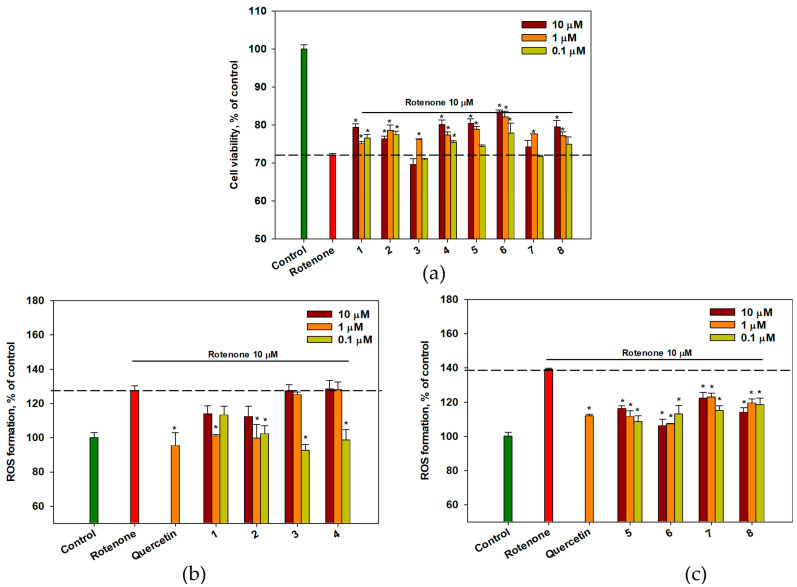
The effect of OSs on cell viability (**a**) and ROS levels (**b**,**c**) in Neuro-2a cells treated with rotenone. Each bar represents the mean ± SEM of three independent experiments. (*) indicates *p* < 0.05, compared with cells treated with rotenone. The dashed line indicates the level of positive control. The green bar represents the control, while the red bar represents the positive control. Differences between the control and rotenone-treated cells were considered significant.

The effect of OSs **1**–**8** on Neuro-2a cell viability in a rotenone-induced neurotoxicity model was investigated (Figure 5a). Rotenone decreased the viability of Neuro-2a cells by 28%. Pre-treatment of the cells with OSs for 1 h prior to the addition of rotenone resulted in an increase in the percentage of living Neuro-2a cells.

Compounds **1** to **8** at concentrations of 0.1 to 10 μM increased the percentage of living Neuro-2a cells after rotenone treatment from 5.8 (0.1 μM) to 14.1% (10 μM) compared to rotenone-treated cells (*p* < 0.05). Compound **6** at concentrations of 10 μM and 1 μM was the most active and increased the viability of rotenone-treated cells by 14.1% and 12.1%, respectively (Figure 5a).

Rotenone significantly increased intracellular ROS levels by 30–40% (Figure 5b,c). All OSs significantly reduced ROS levels in rotenone-treated cells (Figure 5b,c). Compounds **3**, **4**, **5**, and **6** exhibited the highest activity in this assay, resulting in reductions in ROS levels of 27.4 (0.1 μM), 22.8 (0.1 μM), 21.9 (0.1 μM), and 23.6% (10 μM), respectively, compared to cells treated with rotenone. Moreover, these polyphenolic compounds lowered the intracellular level of ROS more effectively than quercetin did (Figure 5b,c).

**Figure 6:**
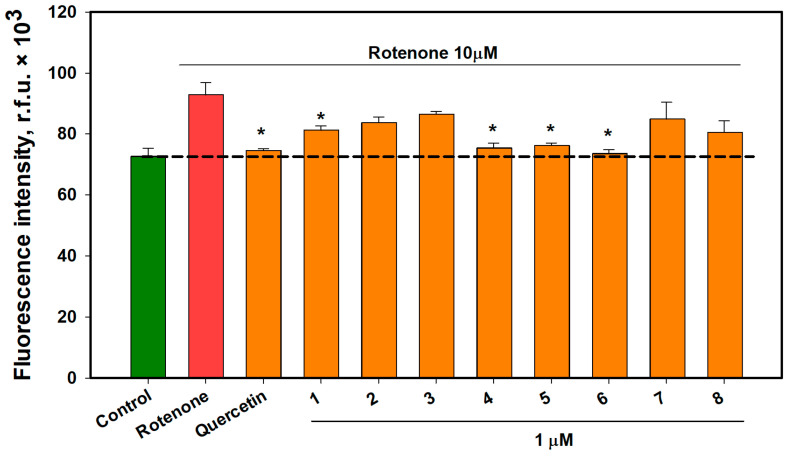
The effect of OSs on mitochondrial ROS levels in Neuro-2a cells treated with rotenone for 1 h. Each bar represents the mean ± SEM of three independent experiments. (*) indicates *p* < 0.05 compared to cells treated with rotenone. The dashed line indicates the level of control. The green bar represents the control, while the red bar represents the positive control. The difference between the control and rotenone-treated cells was considered significant.

The influence of compounds **1**–**8** on mitochondrial ROS levels in rotenone-treated Neuro-2a cells was also studied, and the data are presented in Figure 6.

**Figure 7:**
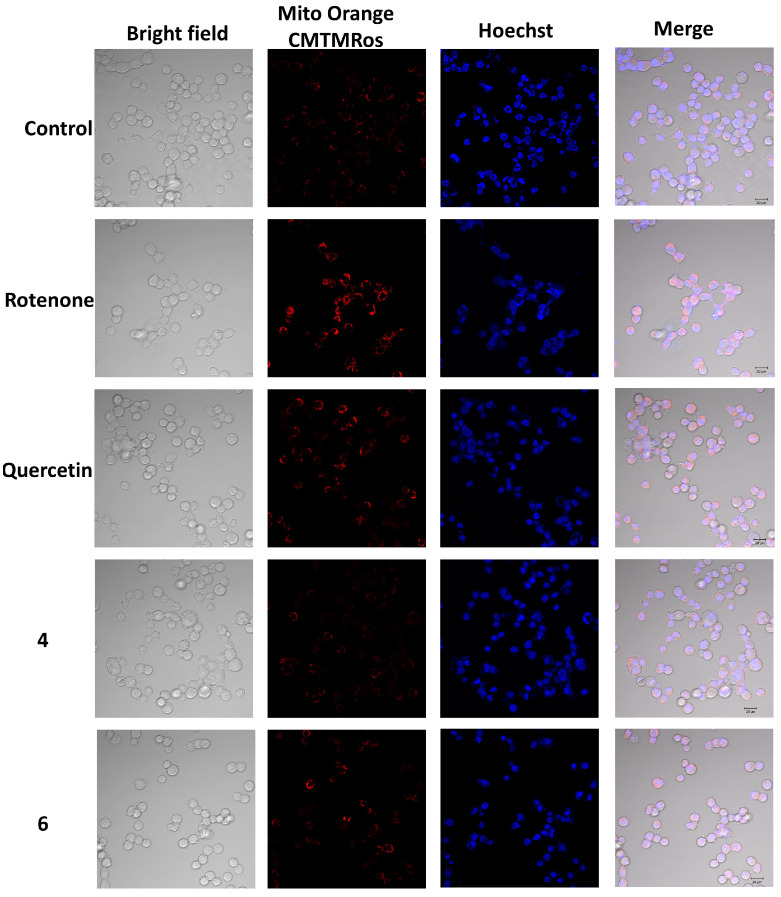
Confocal fluorescence imaging of mitochondrial ROS in Neuro-2a cells treated with rotenone for 1 h. The merged images display three channels: blue DAPI staining for nuclei (excitation at 405 nm), red Mito Orange CM-H2TMRos staining (excitation at 543 nm), and transmitted light detection for a common view. Scale bar = 20 μM.

Rotenone treatment for 1 h increased mitochondrial ROS levels by 27.8%. Compound **1** decreased the mitochondrial ROS level in rotenone-treated cells by 12.5%, and compounds **4**–**6** decreased it by 18.7%, 17.9%, and 20.8%, respectively. Compounds **2**, **3**, **7**, and **8** showed no significant effects in this assay. Thus, vitisin A (**4**) and *trans*-vitisin B (**6**) were the most active and their effects on mitochondrial ROS were visualized using confocal microscopy (Figure 7). The confocal images showed that rotenone increased Mito Orange CM-H2TMRos fluorescence in neuroblastoma cells after incubation for 1 h. At the same time, pre-treatment of cells with substances **4** and **6**, before adding rotenone, significantly reduced the fluorescence intensity of the Mito Orange CM-H2TMRos probe, indicating a decrease in the content of mitochondrial ROS in Neuro-2a cells.

The influence of compounds **1**–**8** on mitochondrial membrane dysfunction induced by rotenone was studied using the potential-dependent fluorescent dye tetramethyl rhodamine ethyl ester (TMRE) to detect MMP, and MitoCLox fluorescent dye was used to detect cardiolipin peroxidation.

**Figure 8:**
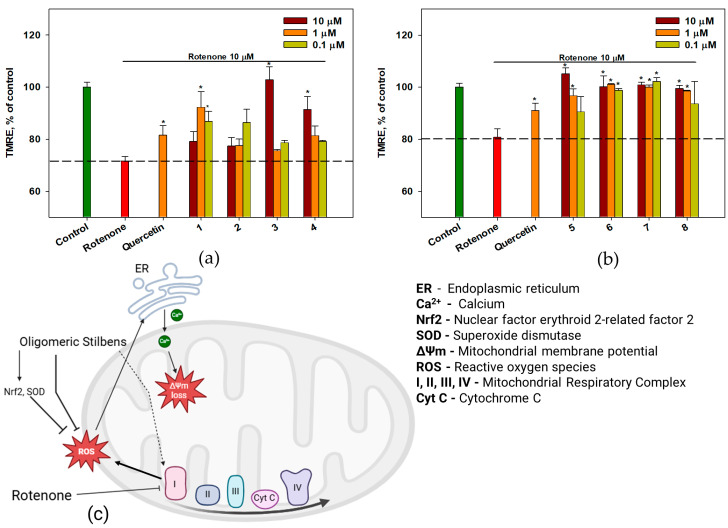
The effect of OSs **1**–**4** (**a**) and **5**–**8** (**b**) on mitochondrial membrane potential in Neuro-2a cells treated with rotenone. The proposed mechanism by which oligomeric stilbenes protect against the rotenone-induced loss of mitochondrial membrane potential (**с**). Each bar represents the mean ± SEM of three independent experiments. (*) indicates *p* < 0.05, compared with cells treated with rotenone. The dashed line indicates the level of positive control. The green bar represents the control, while the red bar represents the positive control. The difference between control and rotenone-treated cells was considered significant. (**с**) was created with Biorender.com.

The exposure of Neuro-2a cells to rotenone for 1 h induced membrane depolarization and decreased TMRE fluorescence intensity by 20–28% (Figure 8a,b). Compounds **1**, **3**, **4**, **5**, **6**, **7**, and **8** increased the mitochondrial membrane potential by 12–43% at different concentrations. Compounds **3** and **5** proved to be the most effective in this analysis, restoring mitochondrial membrane potential values to control levels. The proposed mechanism by which oligomeric stilbenes protect against rotenone-induced loss of mitochondrial membrane potential is shown in Figure 8c.

**Figure 9:**
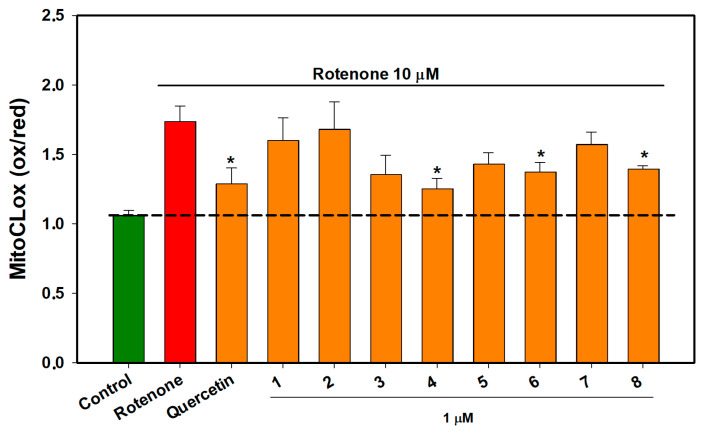
The effect of compounds **1**–**8** on mitochondrial cardiolipin peroxidation in Neuro-2a cells treated with rotenone for 3 h. Each bar represents the mean ± SEM of three independent experiments. (*) indicates *p* < 0.05, compared to cells treated with rotenone. The dashed line indicates the level of control. The green bar represents the control, while the red bar represents the positive control. The difference between the control and rotenone-treated cells was considered significant.

The mitochondrial cardiolipin peroxidation level was measured as a ratio of MitoCLox dye fluorescence at 520 nm and 590 nm, and these data are presented in Figure 9.

Rotenone treatment for 3 h induced a significant increase in the value of the fluorescence ratio 520/590 of MitoCLox dye (1.73) compared to untreated cells (1.06), indicating a strong increase in cardiolipin peroxidation in mitochondrial membranes. Compounds **4**, **6**, and **8,** as well as the reference antioxidant quercetin, restored the values of the 520/590 ratio up to 1.27, 1.25, 1.37, and 1.39, respectively.

### 2.7. Impact of OSs on Superoxide Dismutase (SOD) Activity in Neurotoxin-Treated Cells

As OSs showed weak radical scavenging activity in cell-free assays, it was determined whether the cytoprotective effect of OSs is due to their effect on the antioxidant cellular machinery, including the antioxidant enzyme superoxide dismutase (SOD). The SOD activity in neurotoxin-treated Neuro-2a cells was studied, and the results are presented in Table 2.

The cells were pretreated with compounds at 1 µM, and then neurotoxins 6-OHDA and rotenone were added for 1 or 3 h before the cells were lysed for SOD activity measurement. Thus, both 6-OHDA and rotenone did not affect SOD activity in the cells for 1 h, but dramatically reduced the activity of SOD by 8 or 4 times, respectively, after 3 h of incubation. Pretreatment with compounds **1** and **7** did not enhance SOD activity in 6-OHDA- and rotenone-treated cells after 1 h, but significantly protected enzyme activity after 3 h. In contrast, pretreatment with compound **6** induced a significant restoration and even an increase in SOD activity in 6-OHDA- and rotenone-treated cells after 1 h compared to the control untreated cells. OS **4** also caused a substantial defense of SOD in 6-OHDA-treated cells.

## 3. Discussion

Various neurotoxin-induced cellular models of PD have been used to screen for active substances [27,28]. As the detailed mechanism of PD development at the cellular level remains unclear, it is important to use a combination of modeling approaches. This will allow us to cover different variants of PD development and select substances that are active under different conditions, which will increase the chances of success in the next stage of the investigation. In the present study, neuronal cells were treated with neurotoxins MPP+, PQ, 6-OHDA, and rotenone. All these neurotoxins induce ROS-dependent cell damage, but there are differences in the mechanisms of their toxicity [29].

Despite the fact that we used only one cell line, our results confirm existing trends in the field of research on natural compounds and their effects. It is important to note that none of the studied compounds provided complete protection. We believe that even minor effects can be important for further understanding of the mechanisms of their action and potential therapeutic applications.

The preventive cytoprotective effects of Alpha grape OS ampelopsin A (**1**), ε-viniferin (**2**), vitisin D (**3**), and vitisin A (**4**), as well as α-viniferin (**5**), *trans*-vitisin B (**6**), *cis*-vitisin B (**7**), and melanoxylin A (**8**) against neurotoxin-induced Neuro-2a cell damage were reported in present study. Vitisin D (**3**), vitisin A (**4**), α-viniferin (**5**), *trans*-vitisin B (**6**), and *cis*-vitisin B (**7**) significantly enhanced the viability of PQ- and 6-OHDA-treated Neuro-2a cells. On the other hand, ampelopsin A (**1**), ε-viniferin (**2**), vitisin D (**3**), vitisin A (**4**), and α-viniferin (**5**) were more effective in the MPP+-induced PD model. Finally, all compounds increased the cell viability of the rotenone-treated cells, but only ampelopsin A (**1**), vitisin A (**4**), α-viniferin (**5**), and *trans*-vitisin B (**6**) decreased mitochondrial ROS levels and cardiolipin peroxidation. The combination of these effects makes visitin A (**4**) and *trans*-vitisin B (**6**) more promising anti-PD molecules, showing their pronounced neuroprotective activity in all types of tests.

The biological activity of the same OSs has garnered significant attention in recent years, leading to extensive and innovative studies. Ampelopsin A (**1**) was reported as a sphingosine kinase 1-targeted [30] inducer of apoptosis in MDA-MB-231 breast cancer cells [31] and possessed antifungal activity [32]. Moreover, it has been reported that the central administration of ampelopsin A contributes to increased neurocognitive and neuroprotective effects on intrinsic neuronal excitability and behaviors in a scopolamine-induced dementia mouse model [33]. At the same time, the effect of ampelopsin A (**1**) on α-synuclein aggregation and α-synuclein-induced cytotoxicity was not significant [34] and had weak anti-PGE2 activity in LPS-stimulated human chondrocytes [35]. Thus, the preventive cytoprotective effect of ampelopsin A (**1**) in ROS-dependent neurotoxin-induced neuronal cells was reported for the first time. Obviously, the effect of compound **1** is caused by its direct ROS-scavenging properties, as well as the induction of a high level of SOD activity that may strengthen the antioxidant defense cellular system.

ε-Viniferin (**2**) had an anti-inflammatory effect via the targeting of formyl peptide receptor 1 in human neutrophils [36], and Sirtuin 3 (SIRT3)-mediated neuroprotective effects in Huntington’s disease models and Parkinson’s disease models caused by rotenone administration of SH-SY5Y cells [37]. In a previous study, ε-viniferin (**2**) decreased ROS production and mitochondrial depolarization, and also reduced cell apoptosis. Our experimental data confirm this evidence. However, we did not observe a protective effect of compound **2** in PQ-treated Neuro-2a cells. Moreover, the decrease in mitochondrial ROS levels and the prevention of cardiolipin peroxidation in rotenone-treated cells, as well as the great enhancement effect of SOD activation, were not observed in our experiments with compound **2**, which suggests that there may be limitations to the potential of this compound.

Vitisin D (**3**) was first isolated from *Iris lactea* Pall. var. chinensis [38], and this is the first time that this compound has been isolated from the stem of the Alpha grape variety. It has been reported that vitisin D significantly promotes adipogenesis and increases intracellular lipid accumulation in 3T3-L1 cells [39]. In the present study, we found that vitisin D (**3**) demonstrates radical-scavenging activities and prevents Neuro-2a cell damage caused by all used neurotoxins via ROS level reduction.

Vitisin A (**4**) is a resveratrol tetramer, and its effect on scopolamine-induced impaired learning and memory functions in amnesiac ICR mice was recently reported [40]. The ABTS radical scavenging activity of vitisin A (**4**) was reported more than 20 years ago [41]. It was reported that compound **4** suppresses LPS-induced NF-κB activation in RAW 264.7 cells [42]. The protective effects of vitisin A were investigated in β-amyloid-induced PC12 cells, where vitisin A enhanced cell viability and reduced ROS formation [43] in sodium nitroprusside-induced SH-SY5Y cells [44]. However, the published data on the influence of vitisin A on both intracellular and mitochondrial ROS, as well as SOD activity in PD in vitro models, have not been reported. Therefore, our results appear to be the first in this area.

α-Viniferin (**5**) is a trimeric resveratrol derivative and its anti-inflammatory activities have previously been reported [45]. α-Viniferin suppressed the expression of proinflammatory genes iNOS and COX-2 in the early stage of inflammation by inhibiting Akt (alpha serine/threonine-protein kinase B)/PI3K (Phosphoinositide 3-kinase)-dependent NF-κB activation and inhibiting the production of proinflammatory mediators, NO and PGE2 (Prostaglandin E2), in the late stage by stimulating the Nrf2-mediated HO-1 signaling pathway in LPS-stimulated BV2 microglial cells [46]. Recently, it was reported that α-viniferin (**5**) attenuated motor incoordination and cataleptic behavior in an MPTP-induced mouse model of PD [47]. In this study, the authors investigated the effect of compound **5** on the redox status of neuronal SH-SY5Y cells; however, the influence of compound **5** on intracellular and mitochondrial ROS in PD-like neuronal cells was not measured. Thus, our data on the antioxidant effects of α-viniferin (**5**) in neurotoxin-treated neuronal cells are the first to provide more information on the biological activity of this compound.

*Trans*-vitisin B (**6**) and *cis*-vitisin B (**7**) differ by the configuration of a double bond in stilbene moiety that results in a change in the structure and bioactivity of the isomers. Despite the fact that both compounds weakly increased the viability of PQ-treated Neuro-2a cells, only *trans*-vitisin B (**6**) significantly prevented 6-OHDA- and rotenone-caused cell damage and increased mitochondrial ROS levels and cardiolipin peroxidation. In our assay, it was observed that both compounds **6** and **7** enhanced intracellular SOD activity in 6-OHDA- and rotenone-treated cells, but in different manners. When the cells were pretreated with *trans*-vitisin B (**6**), the SOD activity nearly doubled after 1 h of treatment with neurotoxin. In contrast, pretreatment with *cis*-vitisin B (**7**) induced SOD activity enhancement after only 3 h, which may be too late to prevent the accumulation of cellular and mitochondrial ROS and cell death.

Melanoxylin A (**8**) was isolated as a novel compound from the wood and bark of Cotylelobium melanoxylon and was found to inhibit plasma glucose elevation after sucrose loading in rats and triglyceride elevation after olive oil loading in mice [26]. This is the first report of the isolation of this OS from the Alpha grape stem and its significant antioxidant activity in PD-like neuronal cells.

Antioxidant activity may be the result of both the direct scavenging of free radicals and the activation of the Nrf2-dependent antioxidant system. In the first case, substances ensure their chelation and «decontamination» through direct interaction with free radicals. In the second case, substances interference in the cytoplasmic interaction of the regulatory protein Keap1(Kelch-like ECH-associated protein 1), and the transcriptional factor Nrf2 (Nuclear factor erythroid 2-related factor 2) leads to the translocation of Nrf2 into the nucleus and the initiation of the transcription of genes that encode antioxidant enzymes SOD, HO-1, and others.

In our study, all compounds affected SOD activity in Neuro-2a cells after 3 h of 6-OHDA and rotenone treatment. However, only compounds **4** and **6** or only **6** enhanced SOD activity after 1 h of 6-OHDA and rotenone addition, respectively. This resulted in the more significant neuroprotective activity of compound **6** in PD-like cell models.

Previously, it was reported that a complex root extract of *Vitis vinifera* containing seven stilbenoids, including resveratrol, piceatannol, *trans*-ɛ-viniferin, ampelopsin A, miyabenol C, vitisin A, and vitisin B, protects HT-29 cells against hydrogen peroxide-induced DNA damage and induces Nrf2 and its target genes heme oxygenase-1 and γ-glutamylcysteine synthetase in Huh7 cells [48]. In 2015, visitin B was found to inhibit hepatitis C virus (HCV) replication, but not the Nrf2 activator [49]. In 2023, Kwon et al. [50] reported that vitisin B inhibits neuraminidase activity and suppresses H1N1 viral replication in MDCK and A549 cells, and also decreases virus-induced ROS generation by nuclear translocation of Nrf2 and the enhancement of Nrf2-controlled enzyme activity. Such diametrically opposed conclusions may be related to the specifics of conducting experiments, since in the first study, the authors measured the luminescence level in Nrf2-ARE-luc plasmide-transfected Huh7.5 cells after 48 h and could no longer see a rapid effect.

We have now received some indirect supporting evidence that *trans*-vitisin B activates the Nrf2 machinery in neurodegeneration caused by neurotoxins. PD develops because of genetic disruptions and environmental factors, which may be of particular importance due to genetic disorders. Thus, there is a relationship between certain polymorphisms in the SOD-encoded genes and PD development [51,52]. It was reported that some polymorphism in Nrf2 encoded gene *NFE2L2* (Nuclear Factor Erythroid 2-Related Factor 2) results in reduced resistance of cells to rotenone toxicity [53]. In this regard, dietary antioxidants, including antioxidant defense activators such as OSs, can have a significant impact on curbing the development of PD.

Due to the scarcity of published data concerning the biological activity of certain OSs, such as vitisin D (**3**), *trans*-vitisin B (**6**), and *cis*-vitisin B (**7**), we conducted a comprehensive investigation of these compounds in this study. The objective of our research was to assess the antioxidant and neuroprotective properties of these substances and to juxtapose our findings with those of previously studied OSs. We hypothesized that these compounds also possess notable biological activity and may have potential applications in the prevention and treatment of various diseases linked to oxidative stress and inflammation.

The obtained results showed that all OSs (**1**–**8**) increased cell viability and decreased reactive oxygen species (ROS) levels in the neurotoxin-treated Neuro-2a cells. We found no significant correlations between their structural features and neuroprotective and antioxidant properties. However, among the OSs, only vitisin A (**4**) and *trans*-vitisin B (**6**) demonstrated promising neuroprotective properties, with compound **6** causing the most significant increase in SOD activity in 6-OHDA- and rotenone-treated cells due to the mechanisms proposed in Figure 8c. Rotenone inhibits mitochondrial respiratory complex I, leading to the accumulation of ROS. These ROS can enter the cytoplasm and affect the endoplasmic reticulum, which subsequently causes the release of calcium ions. An increase in calcium levels in the mitochondria can lead to a decrease in mitochondrial membrane potential. Our studies have shown that oligomeric stilbenes influence both Nrf2 and superoxide dismutase, and also directly reduce the levels of reactive oxygen species. This may contribute to the protection of mitochondria from oxidative stress and the preservation of mitochondrial membrane potential. Additionally, one of the potential mechanisms of action of oligomeric stilbenes may involve their effect on mitochondrial respiratory complex I; however, this effect was not demonstrated in our work. Nonetheless, the influence of resveratrol on mitochondrial complex I has been shown in previous studies [54].

We hypothesized that the OSs possess significant biological activity and may have potential applications in the prevention and treatment of various diseases associated with oxidative stress and inflammation. Among these compounds, *trans*-vitisin B (**6**) will be the most promising in future studies in neurotoxin-induced Parkinson’s disease in vivo models.

We believe that our work will contribute to the enhancement of knowledge regarding the biological activity of OSs and inspire further research in this area.

## 4. Materials and Methods

### 4.1. Plant Material

The Alpha grape variety was collected in the south of the Primorsky region of Russia. The herbarium specimen of the Alpha grape variety (voucher No. 267600) was stored in the bioresource collection of the A.K. Chaika Federal Scientific Center of Agrobiotechnology of the Far East (Ussuriisk, Primorsky region, Russia).

### 4.2. Extraction and Isolation

Crushed stems of the grape of Alpha variety (300 g) were extracted twice in a Soxhlet apparatus with the solvent system CHCl_3_–EtOH (3:1) for 3 h at 60 °C. The obtained extract (13 g) of dried stems of the Alpha grape variety was chromatographed on a column packed with polyamide 6DF (Sigma-Aldrich, St. Louis, MI, USA). The column was eluted with the solvent system hexane–CHCl_3_ with a gradual increase in the CHCl_3_ content (fractions 1–8), and then with the solvent system CHCl_3_–EtOH with a gradual increase in the EtOH content (fractions 9–19). Fractions containing stilbenes, according to the HPLC and HPLC-MS data, were selected for further isolation of individual compounds.

Fractions 9 and 10 were chromatographed on a silica gel column (40–63 µ, Sigma-Aldrich, St. Louis, MI, USA), resulting in the isolation of *trans*-vitisin B (**6**) (7.7 mg), *cis*-vitisin B (**7**) (4.5 mg), and α-viniferin (**5**) (4.8 mg). Vitisin A (**4**) (1.4 mg) and ε-viniferin (**2**) (11.3 mg), as well as ampelopsin A (**1**) (14.5 mg), melanoxylin A (**8**) (3.9 mg), and vitisin D (**3**) (1.3 mg) were isolated from fraction 14 after chromatography on a silica gel column (40–63 µ, Sigma-Aldrich, St. Louis, MI, USA). The final purification of the isolated compounds was carried out by chromatography on a sorbent with reversed phase C-18.

### 4.3. General Experimental Procedures

UV spectra were obtained using a UV-1601 PC spectrophotometer (Shimadzu, Kyoto, Japan). ^1^H and ^13^C NMR spectra were obtained in acetone-*d_6_* using a Bruker AVANCE III DRX-700 instrument (Bruker, Karlsruhe, Germany). The optical rotation angles were measured using a Perkin Elmer 343 polarimeter.

### 4.4. Analytical HPLC-UV-MS

High-pressure liquid chromatography was performed using an LCMS-2020 system and a Discovery HS C18 column (150 × 2.1 mm, particle size 3 μm, Supelco Analytical, Bellefonte, PA, USA), a Supelguard Ascentis C18 guard column (2 × 2.1 mm, particle size 3 μm, Supelco Analytical, Bellefonte, PA, USA) in a binary gradient of H_2_O (A): MeCN (B) with an acetic acid concentration of 0.2%. The gradient was as follows: 0–6 min, 25–35% (B); 6–11 min, 35–60% (B); 11–14 min, 60–90% (B); 14–16 min, 90–25% (B); 16–20 min, 25% (B). The flow rate was 0.2 mL/min, and the column temperature was 40 °C. Chromatograms were recorded at 254 nm and 280 nm. Mass spectra were recorded in the electrospray ionization (ESI) mode, recording negative and positive ions (1.50 kV) in the *m*/*z* range of 100–1100, with drying gas N_2_ (10 L/min) and nebulizer gas N_2_ (1.5 L/min), a desolvation line (DL) temperature of 200 °C, and a thermal block at 250 °C. Before analysis, the samples were filtered through a 0.2 μm PTFE syringe filter. The volume of the injected sample was 2 μL.

### 4.5. DPPH Radical Scavenging Assay

The DPPH (2,2-diphenyl-1-picrylhydrazyl) radical scavenging activity of the stilbenes was estimated as described in [55]. The compounds were dissolved in dH_2_O and added to a DPPH solution in MeOH (10^−4^ M) at various concentrations. The mixture was kept in the dark at room temperature for 20 min, and absorption at 517 nm was measured using a Shimadzu UV 1240 spectrophotometer (Shimadzu, Kyoto, Japan). The DPPH radical scavenging effect (%) of the stilbenes was calculated using Equation (1):

(1) $$\mathrm{DPPH}\;\mathrm{scavenging}\;\mathrm{effect},\;\%=\frac{A_{0}-A_{x}}{A_{0}}\times 100$$

*A*_0_ is the optical density of the DPPH solution without stilbenes (blank sample), and *A_x_* is the optical density of the DPPH solution in the presence of various concentrations of stilbenes.

The known antioxidant quercetin was used as the reference compound. All experiments were repeated three times. The half-maximal inhibitory concentration (IC_50_) of the stilbenes was calculated by plotting the DPPH scavenging activity (%) as a function of the concentration. All experiments were carried out in triplicate, and the IC_50_ values are presented as the mean ± standard deviation (SD).

### 4.6. Ferric-Reducing Antioxidant Power Assay (FRAP)

The Ferric-Reducing Antioxidant Power Assay was performed as described previously [56]. The FRAP reagent was prepared by mixing 2.5 mL of 2,4,6-tris(2-pyridyl)-s-triazine (TPTZ) solution (10 mM) in 40 mM of HCl and 25 mL of the FeCl_3_ solution (20 mM) in an acetate buffer (300 mM, pH 3.6). The stilbenes were dissolved in dH_2_O and added to 3 mL of the FRAP reagent at various concentrations. The mixture was then incubated in the dark at room temperature for 4 min. The absorbance was then measured at 595 nm using a Shimadzu UV 1240 spectrophotometer. The ferric-reducing power was calculated using Equation (2):

(2) $$\mathrm{FRAP}=\frac{C_{Fe}}{C_{x}},$$

where *C_Fe_* is the concentration of Fe^2+^ formed in the reaction (μM) and *C_x_* is the concentration of stilbenes in the reaction mixture.

The concentration of Fe^2+^ formed in the reaction (μM) was determined using a calibration curve obtained for different concentrations of FeSO_4_·7H_2_O. All experiments were carried out in triplicate, and data are presented as the mean ± standard deviation (SD).

### 4.7. Cell Line and Culture Conditions

The mouse neuroblastoma cell line, Neuro-2a (CCL-131), was purchased from the American Type Culture Collection (ATCC) (Manassas, VA, USA). Cells were cultured in Dulbecco’s modified Eagle’s medium (Biolot, St. Petersburg, Russia). The medium contained 10% fetal bovine serum (Biolot, St. Petersburg, Russia) and 1% penicillin/streptomycin (Biolot, St. Petersburg, Russia). The cells were incubated at 37 °C in a humidified atmosphere containing 5% (*v*/*v*) CO_2_.

The Neuro-2a cells were seeded in a 96-well plate at a concentration of 2 × 10^4^ cells/well and cultured at 37 °C in a CO_2_ incubator for 24 h before all experiments were started.

### 4.8. In Vitro Model of Neurotoxicity Induced by PQ, Rotenone, MPP+, and 6-OHDA

The neurotoxins 1-methyl-4-phenylpyridine (MPP+), rotenone, 6-hydroxydopamine (6-OHDA), and paraquat (PQ) were used to induce Parkinson’s disease-like cell damage models. PQ (1 mM), MPP+ (1 mM), rotenone (10 μM), and 6-OHDA (120 μM) were used in all the experiments. All neurotoxins were obtained from Sigma-Aldrich, St. Louis, MO, USA. These neurotoxins were used at concentrations considered to be mildly toxic.

The Neuro-2a cells (2 × 10^4^ cells/well) were pretreated with OSs at concentrations of 0.1–10 μM for 1 h. The neurotoxins were then added for the required time. The cells were incubated without any additives or neurotoxins as well as without OSs as the positive and negative controls, respectively.

### 4.9. Cell Viability MTT Assay

Stock solutions of OSs (10 mM) were prepared in dimethyl sulfoxide (DMSO). The solutions of all the tested compounds were diluted in PBS to a volume of 20 μL and added to the wells of the plates at final concentrations of 0.1, 1.0, and 10.0 μM (the final DMSO concentration was <1%). Then, 20 μL of the test solution was loaded onto the cells and incubated for 24 h before the cell viability was measured using the MTT assay.

After incubation, the medium containing OSs was replaced with 100 μL of fresh medium. Next, 10 μL of 3-(4,5-dimethylthiazol-2-yl)-2,5-diphenyltetrazolium bromide (MTT) stock solution (Sigma-Aldrich, St. Louis, MO, USA) (5 mg/mL) was added to each well, and the microplate was incubated for 4 h. Then, 100 μL of the SDS-HCl solution (1 g SDS/10 mL dH_2_O/17 μL 6 M HCl) was added to each well, followed by incubation for 18 h. The absorbance of the converted formazan dye was measured at 570 nm using a Multiskan FC microplate reader (Thermo Scientific, Waltham, MA, USA) at 570 nm [57]. All experiments were performed in triplicate, and the cytotoxic activity was expressed as the percentage of living cells.

### 4.10. Assessment of Intracellular Reactive Oxygen Species (ROS) Levels

Neuro-2a cells (2 × 10^4^ cells/well) were preincubated with OS at concentrations of 0.1–10 μM for 1 h, and then rotenone (10 μM), 6-OHDA (120 μM), or PQ (1 mM) were added for 1 h, 1 h, or 3 h, respectively. To assess the intracellular ROS levels, we added 20 μL of 2,7-dichlorodihydrofluorescein diacetate solution (10 μM, H2DCF-DA, Molecular Probes, Eugene, OR, USA) to each well to obtain a final concentration of 10 μM, and incubated the microplate at 37 °C for an additional 30 min. Quercetin was used as the reference compound. After incubation, the fluorescence intensity at λex = 540 nm and λem = 590 nm was measured using a PHERAstar FS plate reader (BMG Labtech, Ortenberg, Germany), and the data were processed using MARS Data Analysis v3.01R2 (BMG Labtech, Ortenberg, Germany) as a percentage of the positive control value.

### 4.11. Mitochondrial ROS Level Assay

The specific fluorescent dye LumiTracker^®^ Mito Orange CM-H2TMRos (Lumiprobe, Moscow, Russia), which is a reduced non-fluorescent version of the CMTMRos dye that becomes fluorescent upon oxidation on the mitochondrial membrane, was used to measure mitochondrial ROS levels.

Neuro-2a cells (2 × 10^4^ cells/well) were preincubated with OSs at concentrations of 0.1–10 μM for 1 h and then rotenone (10 μM), 6-OHDA (120 μM), or PQ (1 mM) were added for 1 h, 1 h, or 3 h. Then, the cells were washed with PBS, and Mito Orange CM-H2TMRos dissolved in PBS at 100 nM was added to each well for 1 h. After this, the cells were washed twice with PBS. The fluorescence intensity was measured at λex = 540 nm and λem = 590 nm, and the data were processed using MARS Data Analysis v3.01R2 (BMG Labtech, Ortenberg, Germany). The results are presented as a percentage of the positive control value.

Moreover, other cell samples were additionally stained with DAPI (300 nM for 5 min) and visualized using confocal microscopy. Laser Confocal Imaging was performed using LSM 710 LIVE scanning confocal laser microscopes (Carl Zeiss, Jena, Germany) with an excitation wavelength of 543 nm and an emission filter of 570–670 nm for Mito Orange CM-H2TMRos dye. DAPI was excited at 405 nm and captured using a 450/50 nm bandpass filter. The confocal photos were processed using LSM 510 Release version 4.2 and the ZEN 2011 software.

### 4.12. Determination of Mitochondrial Membrane Potential (MMP)

Neuro-2a cells (2 × 10^4^ cells/well) were preincubated with OSs at concentrations of 0.1–10 μM for 1 h, and then rotenone (10 μM) was added for 1 h. Cells incubated without rotenone and the compounds were used as the positive controls, and cells treated with rotenone alone were used as the negative controls. The fluorescent dye tetramethylrhodamine ethyl (TMRE) solution (Lumiprobe, Moscow, Russia) (250 nM) was added to each well for 30 min at 37 °C, and the cells were washed three times. The fluorescence intensity was measured using a PHERAstar FS plate reader (BMG Labtech, Ortenberg, Germany) at λex = 540 nm and λem = 590 nm. The data were processed using MARS Data Analysis v3.01R2 (BMG Labtech, Ortenberg, Germany) and presented as a percentage of the positive control value.

### 4.13. Cardiolipin Peroxidation Assay

The fluorescent dye MitoCLox (Lumiprobe, Moscow, Russia) was used to measure cardiolipin oxidation in the mitochondria [58], which was detected either as a decrease in absorption at 588 nm or an increase in fluorescence emission in the ratiometric mode at 520/590 nm [59].

MitoCLox was dissolved in DMSO at a concentration of 50 µM and added to the cells (final concentration of 200 nM) for 1 h. After 1 h, the cells were washed with PBS and the fluorescence was measured in the ratiometric mode at λem = 520/590 nm (ox/red ratio) using a PHERAStar FS plate reader (BMG Labtech, Offenburg, Germany). The data were processed using MARS Data Analysis v. 3.01R2 (BMG Labtech, Offenburg, Germany) and calculated as a 520/590 ratio.

### 4.14. Superoxide Dismutase Activity Detection

Neuro-2a cells were seeded in 6-well plates and incubated for 24 h. 6-OHDA or rotenone in dH_2_O was added at concentrations of 120 μM and 10 μM, respectively, (30 μL of each solution) for 1 h, followed by the addition of compounds at a concentration of 1 μM for 1 or 3 h. Untreated cells were used as controls. The cells were washed twice with PBS and lysed with an RIPA buffer (Sigma-Aldrich, St. Louis, MO, USA) using a triple freeze–defrost cycle. The lysates were collected in 1.5 mL tubes and centrifuged at 14,000 rpm for 10 min (Eppendorf, Framingham, MA, USA). After this, the supernatant was used for further experiments. The reaction mixture contained 1 mL of 26.9 μM EDTA, 1 mL of 4.04 μM nitroblue tetrazolium chloride (Dia-M, Novosibirsk, Russia), 1 mL of 65 μM 5-methylphenazinium methyl sulfate (Dia-M, Novosibirsk, Russia), and 26 mL of PBS. The supernatant, 1 mM NADH, and reaction mixture were added to a 96-well plate at a ratio of 1:1:28, incubated for 10 min in the dark, and the reaction was stopped in the light. The mixture with the RIPA buffer instead of the supernatant was used as the control. A mixture without NADH was used as the background. The optical densities of the reaction mixtures were measured at λ = 540 nm using a PHERAstar FS plate reader (BMG Labtech, Ortenberg, Germany).

The superoxide dismutase activity (Asod, U/mg) was determined by the inhibition of nitroblue tetrazolium reduction (T, %) and optical density (OD):

T (%) = (control OD − test OD)/control OD × 100%, and this was calculated for the total protein content.

The total protein content was measured using the Bradford method. Bovine serum albumin was used to construct a calibration curve.

### 4.15. Statistical Analysis

All experiments were performed in triplicate and Student’s *t*-test was performed using SigmaPlot 14.0 (Systat Software Inc., San Jose, CA, USA) to determine statistical significance.

## 5. Conclusions

This study is related to the investigation of the neuroprotective and antioxidant properties of OSs from Alpha grape stems, including ampelopsin A, ε-viniferin, vitisin D, vitisin A, α-viniferin, *trans*-vitisin B, *cis*-vitisin B, and melanoxylin A. Our findings demonstrate the significant cytoprotective effects of the studied compounds against neurotoxin-induced damage in Neuro-2a neuronal cells, suggesting their potential as therapeutic agents for Parkinson’s disease (PD).

In particular, vitisin A and *trans*-vitisin B showed robust neuroprotective effects and enhanced antioxidant defenses by increasing superoxide dismutase activity. This highlights the need for multifaceted approaches in PD research to better understand neuronal damage mechanisms and to identify effective treatments.

Given the limited data on some OSs, our findings provide valuable insights into their roles in neuroprotection and oxidative stress management. Further research should further explore their mechanisms and efficacy in vivo.

## Figures and Tables

**Table 1:** DPPH radical-scavenging and ferric-reducing activity of OSs.

Comp	1	2	3	4	5	6	7	8	Quercetin
DPPH, IC_50_ µM	717.5 ± 26.6 *	63.0 ± 1.1 *	62.7 ± 0.63 *	59.1 ± 2.5 *	409.0 ± 41.4 *	314.0 ± 33.2 *	333.8± 36.6 *	528.9 ± 56.5 *	9.5 ± 0.5
FRAP, µM	0.56 ± 0.06 *	1.48 ± 0.13 *	0.76 ± 0.08 *	0.71 ± 0.07 *	0.66 ± 0.07 *	0.34 ± 0.03 *	0.99 ± 0.12 *	1.10 ± 0.14 *	3.30 ± 0.23

IC50 (Half Maximal Inhibitory Concentration). DPPH (2,2-diphenyl-1-picrylhydrazyl). FRAP (Ferric-Reducing Antioxidant Power Assay). Data are presented as the mean values ± SD, n = 3, (*) indicates *p* < 0.05 compared to quercetin.

**Table 2:** Influence of OSs on SOD activity in neurotoxin-treated Neuro-2a cells.

Compound	6-OHDA		Rotenone	
	1 h	3 h	1 h	3 h
control	9.43 ± 0.78	7.29 ± 1.15	8.75 ± 2.27	7.74 ± 2.38
neurotoxin	8.69 ± 0.48	0.92 ± 0.28 #	8.69 ± 1.93	2.10 ± 0.34 #
**1**	10.47 ± 2.40	7.24 ± 2.44 *	6.73 ± 4.25	8.68 ± 2.33 *
**2**	8.88 ± 0.46	4.93 ± 1.13 *	6.06 ± 3.73	5.92 ± 2.35
**3**	7.24 ± 5.58	6.80 ± 4.24	4.63 ± 1.15	8.16 ± 1.09 *
**4**	12.84 ± 2.72 *	5.51 ± 1.37 *	4.10 ± 1.34	6.6 ± 2.65 *
**5**	3.51 ± 0.80	4.59 ± 0.23 *	8.64 ± 0.87	5.50 ± 1.28
**6**	14.21 ± 5.03 *	2.57 ± 1.49	19.94 ± 1.33 *	3.09 ± 0.79
**7**	5.42 ± 0.45	7.24 ± 4.44 *	6.73 ± 4.25	8.68 ± 2.33 *
**8**	5.27 ± 5.51	2.06 ± 2.16	7.66 ± 1.41	6.47 ± 1.59 *

Data are presented as the mean values ± SE, n = 3. (*) indicates *p* < 0.05 compared to cells treated with 6-OHDA or rotenone; (#) indicates *p* < 0.05 compared to control untreated cells.

## Data Availability

The data are contained within the article and Supplementary Materials. References [24,25,26] are cited in the Supplementary Materials.

## Supplementary Materials

The following supporting information can be downloaded at https://www.mdpi.com/article/10.3390/ijms26062411/s1.
