# Supplementary material for: Neuroprotective Activity of Oligomeric Stilbenes from Alpha Grape Stems in In Vitro Models of Parkinson’s Disease
Source: Int J Mol Sci. 2025 Mar 7;26(6):2411. doi: 10.3390/ijms26062411 (PMC11942555; doi:10.3390/ijms26062411)
Supplement: Supplementary file 1 [file ijms-26-02411-s001.zip › ijms-3474492-supplementary.pdf]

Supplementary data

Neuroprotective Activity of Oligomeric Stilbenes from Alpha Grape Stems in In Vitro Models of Parkinson's Disease

Evgeny A. Pislyagin , Darya V. Tarbeeva , Ekaterina A. Yurchenko , Ekaterina S. Menchinskaya, Tatiana Y. Gorpenchenko, Natalya D. Pokhilo , Anatoly I. Kalinovskiy , Dmitry L. Aminin and Sergey A. Fedoreyev

The chemical characteristics of compounds **5**-**8**.

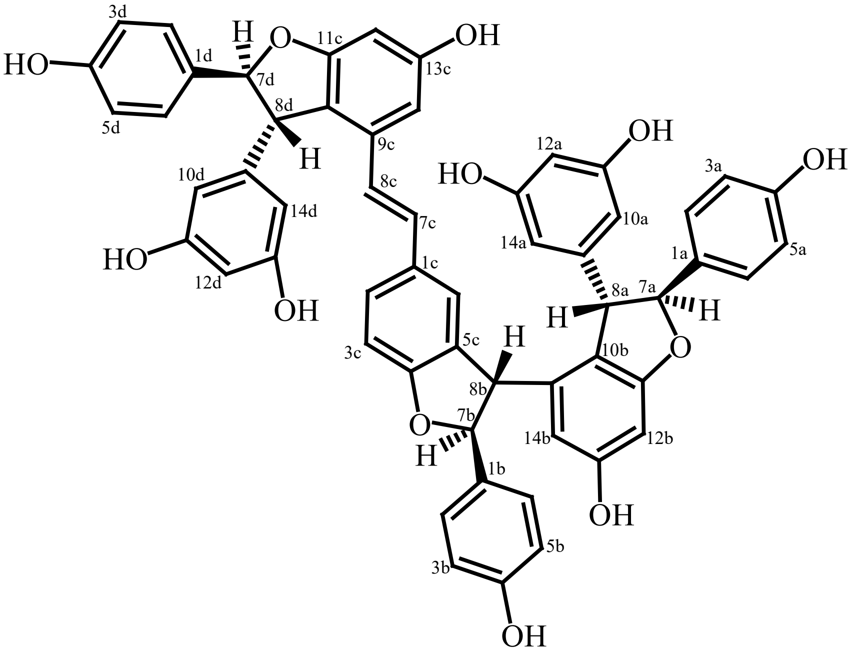

Trans-vitisin B (**6**)

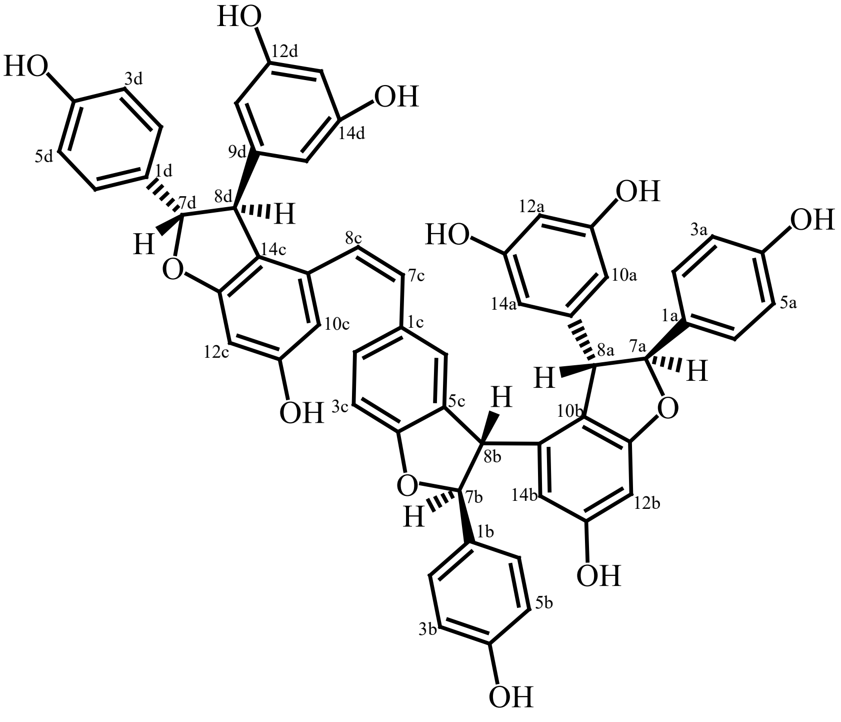

Cis-vitisin B (**7**)

α-Viniferin (**5**): white, amorphous powder; UV (EtOH) λ_{max} 198, 286 nm; α_D^{20} = – 44°; ^{1}H and ^{13}C NMR spectra were in accordance with previously published data [21]; ESI-MS *m/z* 905 [M-H]$^-$, *m/z* 907 [M+H]$^+$.

Trans-vitisin B (**6**): white, amorphous powder; UV (EtOH) λ_{max} 227, 323 nm; α_D^{20} = – 114°; ^{1}H NMR 7.25 (2H, dd, J = 8.6, 1.9 Hz, H-2a, 6a), 7.20 (2H, dd, J = 8.5, 2.0 Hz, H-2d, 6d), 7.13 (1H, dd, J = 8.4, 1.8 Hz, H-2c), 6.92 (2H, dd, J = 8.6, 1.9 Hz, H-3a, 5a), 6.82 (1H, d, J = 1.8 Hz, H-6c), 6.81 (2H, dd, J = 8.5, 2.0 Hz, H-3d, 5d), 6.74 (1H, d, J = 8.4 Hz, H-3c), 6.73 (1H, d, J = 16.1 Hz, H-8c), 6.67 (1H, d, J = 2.0 Hz, H-14c), 6.62 (2H, dd, J = 8.5, 1.9 Hz, H-2b, 6b), 6.61 (1H, d, J = 16.1 Hz, H-7c), 6.58 (2H, dd, J = 8.5, 1.9 Hz, H-3b, 5b), 6.31 (1H, d, J = 2.1 Hz, H-12b), 6.30 (1H, d, J = 2.0 Hz, H-12c), 6.23 (1H, t, J = 2.1 Hz, H-12d), 6.20 (3H, m, H-12a, 10d, 14d), 6.19 (1H, d, J = 2.1 Hz, H-14b), 6.11 (2H, d, J = 2.2 Hz, H-10a, 14a), 5.53 (1H, d, J = 5.1 Hz, H-7b), 5.40 (1H, d, J = 5.5 Hz, H-7a), 5.38 (1H, d, J = 5.4 Hz, H-7d), 4.52 (1H, d, J = 5.5 Hz, H-8a), 4.45 (1H, d, J = 5.4 Hz, H-8d), 4.32 (1H, d, J = 5.1 Hz, H-8b); ^{13}C NMR 162.5 (C-11c), 162.4 (C-11b), 159.8 (C-11a, 13a, 13b, 4c, 11d, 13d), 159.3 (C-13c), 158.1 (C-4a, 4d), 157.8 (C-4b), 147.4 (C-9a, 9d), 142.1 (C-9b), 136.4 (C-9c), 134.1 (C-1a), 133.9 (C-1d), 132.1 (C-5c), 131.8 (C-1b), 131.7 (C-1c), 130.3 (C-8c), 127.9 (C-2d, 6d),127.8 (C-2a, 6a), 127.6 (C-2b, 6b), 126.2 (C-2c), 125.7 (C-6c), 124.4 (C-7c), 119.9 (C-10b), 119.8 (C-10c), 116.2 (C-3a, 5a), 115.9 (C-3d, 5d), 115.8 (C-3b, 5b), 110.5 (C-3c), 107.0 (C-14b), 106.9 (C-10a, 14a), 106.8 (C-10d, 14d), 104.6 (C-14c), 102.1 (C-12a), 102.0 (C-12d), 96.9 (C-12b), 96.3 (C-12c), 94.1 (C-7a), 93.9 (C-7d), 91.3 (C-7b), 57.1 (C-8d), 56.9 (C-8a), 52.2 (C-8b); ESI-MS *m/z* 905 [M-H]$^-$, *m/z* 907 [M+H]$^+$[22].

Cis-vitisin B (**7**): white, amorphous powder; UV (EtOH) λ_{max} 226, 285 nm; α_D^{20} = – 28°; ^{1}H NMR 7.20 (2H, dd, J = 8.6, 2.0 Hz, H-2a, 6a), 7.10 (2H, dd, J = 8.6, 2.0 Hz, H-2d, 6d), 7.00 (1H, dd, J = 8.4, 1.5 Hz, H-2c), 6.83 (2H, dd, J = 8.6, 2.0 Hz, H-3a, 5a), 6.82 (2H, dd, J = 8.6, 2.0 Hz, H-3d, 5d), 6.70 (1H, d, J = 1.5 Hz, H-6c), 6.63 (2H, dd, J = 8.7, 1.9 Hz, H-2b, 6b), 6.62 (2H, dd, J = 8.7, 1.9 Hz, H-3b, 5b), 6.60 (1H, d, J = 8.4 Hz, H-3c), 6.31 (1H, d, J = 2.1 Hz, H-12b), 6.28 (1H, d, J = 2.0 Hz, H-12c), 6.26 (1H, d, J = 2.0 Hz, H-14c), 6.23 (1H, t, J = 2.1 Hz, H-12a), 6.20 (1H, d, J = 2.1 Hz, H-14b), 6.19 (1H, t, J = 2.0 Hz, H-12d), 6.10 (2H, d, J = 2.1 Hz, H-10a, 14a), 6.06 (1H, d, J = 12.1 Hz, H-7c), 6.04 (2H, t, J = 2.0 Hz, H-10d,14d), 5.96 (1H, d, J = 12.1 Hz, H-8c), 5.54 (1H, d, J = 4.9 Hz, H-7b), 5.37 (1H, d, J = 4.8 Hz, H-7a), 5.30 (1H, d, J = 5.4 Hz, H-7d),

4.45 (1H, d, J = 4.8 Hz, H-8a), 4.30 (1H, d, J = 4.9 Hz, H-8b), 4.04 (1H, d, J = 5.4 Hz, H-8d); ^{13}C NMR 162.6 (C-11c), 162.3 (C-11b),160.2 (C-13b), 160.1 (C-11a, 13a), 159.3 (C-13c), 159.2 (C-4c), 159.6 (C-11d, 13d), 158.2 (C-4a, 4d), 157.8 (C-4b), 147.3 (C-9a), 147.0 (C-9d), 142.3 (C-9b), 137.1 (C-9c), 133.9 (C-1a), 133.8 (C-1d), 132.4 (C-1b), 132.2 (C-5c), 131.1 (C-7c), 131.0 (C-1c), 128.1 (C-2d, 6d), 128.0 (C-2a, 6a), 129.5 (C-2c), 127.4 (C-2b, 6b), 127.2 (C-6c),

126.2 (C-8c), 120.0 (C-10c), 119.9 (C-10b), 116.3 (C-3d, 5d), 116.2 (C-3a, 5a), 116.0 (C-3b, 5b), 110.0 (C-3c), 108.4 (C-14c), 107.0 (C-10a, 14a), 106.9 (C-10d, 14d), 106.8 (C-14b), 102.4 (C-12a), 102.0 (C-12d), 96.8 (C-12c), 96.5 (C-12b), 94.2 (C-7d), 94.1 (C-7a), 91.3 (C-7b), 57.1 (C-8d), 56.9 (C-8a), 52.4 (C-8b); ESI-MS *m/z* 905 [M-H]$^-$, *m/z* 907 [M+H]$^+$ [22].

Melanoxylin A (**8**): white, amorphous powder; UV (EtOH) λ_{max} 283 nm; α_D^{20} = – 33°; ^{1}H NMR and ^{13}C NMR spectra were published in [23]; ESI-MS *m/z* 453 [M-H]$^-$, *m/z* 455 [M+H]$^+$.
